# Characterizing the food environment in Scotland and its association with deprivation: A national study

**DOI:** 10.1016/j.pmedr.2025.103254

**Published:** 2025-09-23

**Authors:** Deksha Kapoor, Joe Kennedy, Kirsteen Shields, Christian Reynolds, Tom Clemens, Lindsay M. Jaacks

**Affiliations:** a Division of Global Agriculture and Food Systems, University of Edinburgh, Midlothian, UK; b Centre for Food Policy, City St Georges, University of London, London, UK; c School of Geosciences, University of Edinburgh, Midlothian, UK

**Keywords:** Food environment, Area deprivation, UK, Supermarket, Takeaway, Fast food, Out of home, Retail

## Abstract

**Objectives:**

To characterize food outlets across Scotland and analyze their distribution by neighborhood deprivation.

**Methods:**

Data from the Food Standards Agency of all registered food businesses in 2024 were categorized as out-of-home (OOH) [including restaurants, pubs, cafés, and takeaways], retail [supermarkets and non-food retailers like pharmacies with limited food items] or other [mobile caterers, charity organizations, and home caterers]. Neighborhood deprivation was quantified using the Scottish Index of Multiple Deprivation.

**Results:**

Of all food outlets, 59 % (*n* = 18,409) were OOH, 28 % (*n* = 8757) retail, and 13 % (*n* = 3969) other. The density of OOH (1.9 per km^2^) was more than double that of retail (0.8 per km^2^). Glasgow City had the highest OOH outlet density (18.5 per km^2^). Argyll and Bute, Western Isles, and Highlands had the lowest density of both OOH and retail (≤0.03 per km^2^). Compared to the most deprived neighborhoods, the least deprived neighborhoods had more Restaurants/cafés/canteens (37 % versus 23 % of food outlets, respectively) and fewer Takeaways/sandwich shops (16 % versus 24 % of food outlets, respectively).

**Conclusion:**

Though OOH outlets far outnumber retail in all of Scotland, unique food environments exist in different local authorities. These insights can inform local development and support targeted strategies to improve food environments.

## Highlights

• Comprehensive analysis of food outlets and neighborhood deprivation in Scotland.
• Novel approach of combining text similarity matching with the GPT-4 language model.
• 60 % of outlets were out of home, with their density more than double that of retail.
• Least deprived areas had more restaurants and fewer takeaways than most deprived.

## 1 Introduction

Scotland has the lowest life expectancy at birth of any Western European country and it has continued to fall in recent years (European Union Life expectancy at birth by sex 2024, 2025; *Scottish Government Life Expectancy in Scotland 2020–2022 Provisional Figures*, 2023). The gap in time spent living in good health between individuals in the most and least deprived areas is widening, reaching 24 years in 2019 (David Finch and Bibby, 2023). Disparities in healthy weight likely contribute to healthy life expectancy disparities: 39 % of adults in Scotland's least deprived areas have a healthy weight compared to 28 % of adults in the most deprived areas (Scottish Government, 2022). A Scotland where everyone eats well and has a healthy weight is a key public health priority (Scottish Government, 2018), and several policies have been adopted in recent years to support this vision. These policies have targeted improvements in food environments recognizing their role in influencing what people eat and drink (Katrine Lindberg Hansen SG, 2022). Policies such as taxing unhealthy foods, restricting their promotion, and implementing front-of-pack labeling have been prioritized by governments worldwide including the UK government (Vandevijvere et al., 2019). These policies are generally supported by the public (Adams et al., 2021), and have led to improvements in diet quality, such as reduction in sugar consumption as a result of the Soft Drinks Levy (Scarborough et al., 2020). Several studies in the UK have shown a positive association between proximity (Libuy et al., 2024) and density of fast-food outlets and obesity (Albalawi et al., 2020); though not all studies have shown a significant effect (Green et al., 2021).

In Scotland, the Good Food Nation (Scotland) Act, passed into Scots Law in 2022, aims to ensure that everyone in Scotland has “ready access to the healthy, nutritious food they need” and “People who serve and sell food – from schools to hospitals, retailers, cafes and restaurants – are committed to serving and selling good food”. Hence, all foods prepared outside home at establishments such as restaurants, pubs, cafés, and takeaways are considered out-of-home (OOH). Supporting this vision, Public Health Scotland and Food Standards Scotland (FSS) have developed an Eating Out, Eating Well Framework to help the OOH sector provide healthier foods, recognizing that OOH food and drink tends to be less healthy (Huang et al., 2021). According to FSS's latest report on monitoring OOH food and drink purchases in Scotland, 98 % of the population visited OOH venues in 2023 (Clodagh Elrick NFaLW, 2024).

Differences in food environments may contribute to health disparities and while government policies aim to improve food environments, no study has comprehensively evaluated the food environment across Scotland. To date, studies have only been conducted in Glasgow, Scotland's largest city, and reported a positive association between the density of food outlets and neighborhood deprivation (Macdonald et al., 2018; Sauveplane-Stirling et al., 2014). We, therefore, aimed to (European Union Life expectancy at birth by sex 2024, 2025) characterize the food environment across Scotland and (*Scottish Government Life Expectancy in Scotland 2020–2022 Provisional Figures*, 2023) explore how the food environment varies by neighborhood deprivation.

## 2 Methods

### 2.1 Data source and food outlet classification

**Fig. 1:**
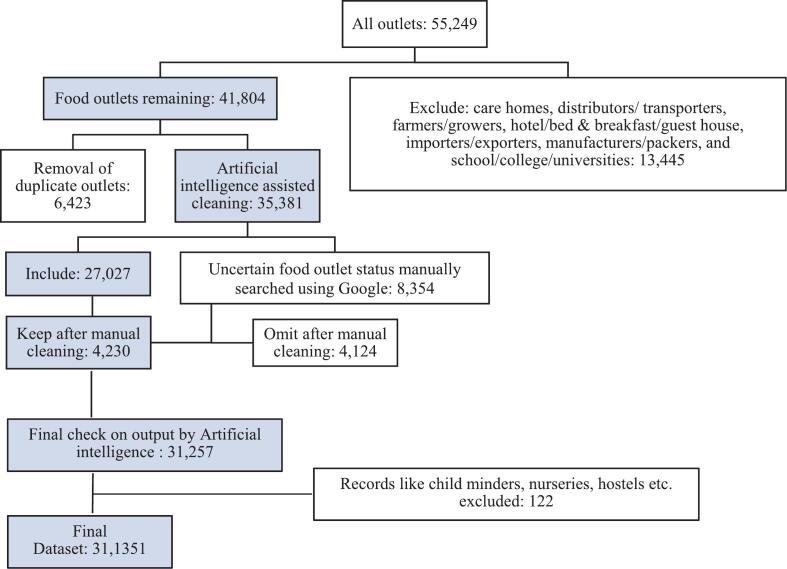
Flowchart of data cleaning of food businesses in Scotland, 2024. ^1^ Outlets with missing postcodes were excluded from Scottish Index of Multiple Deprivation analysis (*n* = 732).

Scotland is divided into 32 designated council areas or local authorities. All food outlets must have a food hygiene inspection, undertaken by the local authority under the Food Hygiene Information Scheme rating. We obtained a list of all food outlets from the UK food hygiene rating data (Scotland) in 2024 (*n* = 55,249), compiled by the Food Standards Agency (FSA): https://ratings.food.gov.uk/open-data, downloaded on 1st February 2024. Data available for each food outlet included business name, address, local authority, geocode, and classification. There were 14 classifications. Seven classifications were excluded from this analysis: care homes, distributors/ transporters, farmers/growers, hotel/bed & breakfast/guest house, importers/exporters, manufacturers/packers, and school/college/universities. From the list of 55,249 food businesses, 13,445 (24 %) were classified in one of these seven categories and excluded, thus 41,804 (76 %) were included in this study (Fig. 1).

We categorized outlets using the existing FSA classifications: OOH (including restaurants, pubs, cafés, and takeaways), retail (including supermarkets and other establishments that primarily sell non-food products and a limited range of food products such as pharmacies), or other (mobile caterers, charity organizations, and home caterers) (Table 1).

**Table 1:** Definition of food outlets in Scotland, 2024.

Food Standards Agency classification^1^	Food Standards Agency description	Categorization for current analysis
Pub/ bar/ nightclub	Commercial establishments that primarily serve alcohol in a public bar. If the establishment has a separate restaurant facility it is recorded under the pub category. Examples: Public Houses, Night clubs/ clubs with bars.	Out of home
Restaurant/ Café/ Canteen	Establishments whose primary business is to cook/ prepare food for customers. Examples: Restaurants, Cafés, Self-service caterer, “Fast food” establishments providing seating, e.g., McDonalds, Burger King, etc. The drive-thru variants of these chains are also included in this category.	
Takeaway/ sandwich shop	Establishments that provide convenience food to customers, primarily for consumption off the premises. Establishments must be immobile and housed in a designated building. These establishments prepare and deliver convenience food directly to the customer. Examples: Fish & chip shops, Takeaway Sandwich shops.	
Retailers - supermarkets/ hypermarkets^2^	Supermarkets. Examples: Sainsbury, Tesco, Asda, Morrison, Co-op, Marks and Spencer, Waitrose, Aldi, Lidl, etc. that provide a range of food items from more than one grocery sector and from a range of brands. City center or local variants of larger supermarket groups, e.g., Sainsbury's local, Tesco Metro, Tesco Express, etc. Establishments like Londis and Spar are also included in this category.	Retail
Retailers - other	Retail establishments which do not fit into one of the other retailer categories, e.g., establishments that primarily sell non-food products and a very limited range of food products. Examples: Shops where the main business is not food for example a chemist/pharmacy. Smaller-scale food businesses such as Grocers, Confectioners, Butchers (retail only), Fishmongers, Greengrocer/fruiterer, Health food shops, Bakers shops (retail only), Newsagents and Garage minimarkets. Establishments like Costcutter, One-Stop, Nisa, and Premier are also included in this category.	
Other catering premises	Restaurant/catering establishments that do not fit into one of the other restaurants and caterers' categories. Examples: Home caterers such as cake makers selling directly to consumers, village halls, community centers, etc. used by charitable/community organizations.	Other
Mobile caterer^3^	A food establishment that comprises a kitchen or catering facility operating from a mobile unit such as a vehicle, trailer, stall, marquee or other non-permanent structure. Examples: Mobile catering units, Burger vans and other fast-food vans/trailers/stalls.	

1 The following business classifications were excluded from this analysis: care homes, distributors/ transporters, farmers/growers, hotel/bed & breakfast/guest house, importers/exporters, manufacturers/packers, and school/college/universities.

2 Cafés in supermarkets are included separately in out of home as Restaurant/Café/Canteen.

3 Mobile caterers were excluded from the out of home category because the address listed in the database reflects the business's registration location, which may differ from the actual site where food is sold.

Since businesses self-selected their classification from the FSA classifications (Table 1), a consistent definition was not always applied across businesses. If an outlet was categorized in more than one way, it appeared multiple times in the data as different records. For example, Costa Coffee at Edinburgh Airport, was categorized twice as “Restaurant/Cafe/Canteen” and “Takeaway/sandwich shop”. The name and address of such outlets were manually reviewed, resulting in removal of 6423 duplicates. The most recent data entry was retained**.**

In addition, some businesses that are not traditionally considered part of OOH or retail, for example, banks and schools, were present in the dataset. The process of removing these non-food businesses was facilitated using Open-AI's GPT-4 large language model (OpenAI JA et al., 2024), by developing an input prompt to identify them based on its domain knowledge and the business description (**Supplementary Material S1**). The model identified 8354 (24 %) potential businesses that should be excluded from the list of OOH and retail outlets. Of these, 34 (0.4 %) were not in the original list and were “hallucinated” by the model, although in many instances these businesses differed by only minor changes in spelling or punctuation from a business in the original list (**Supplementary Material S1**). In addition, this list of potential exclusions contained businesses that could not easily be classified using the name alone such as “A & A Bros” or “1841”. These were manually verified using a Google search. The business name and postcode were searched, typically yielding results such as the outlet's website or its menu on online food delivery platforms. For non-food businesses, details about the company's operations were often displayed. A total of 4124 outlets were manually removed following a Google search. A final manual check was carried out on the list of 31,257 outlets flagged as food outlets from GPT-4 assisted cleaning. This resulted in the removal of 122 records (0.4 %) incorrectly classified as food businesses, including, for example, childminders, nurseries, hostels, etc. The final dataset for analysis included 31,135 food outlets. This novel approach of combining string similarity matching with GPT-4 saved time, improved efficiency, and was found to be accurate through manual validation (only 0.4 % of outlets were misclassified as food outlets).

The “Other catering premises” and “Other retail” contained outlets meeting the definition of other outlet categorizations and so were recategorized accordingly. For example, many outlets with “grill,” “bar,” or “bakes” in the name were recategorized from the “Other catering premises” to “Restaurant/café/canteen”. “Greggs” and outlets with “ice cream” in the name were recategorized to “Takeaway/sandwich shop”. Likewise, outlets like Londis, Morrisons, etc. were recategorized from “Other retail” to “Retailers - supermarkets/hypermarkets”.

This study used publicly available, non-human data and thus does not require ethics approval.

Cleaning was done using string matching in R version 4.2 (R Core Team, 2022).

### 2.2 Data analysis

The density per square kilometer and proportion of OOH, food retail, and other outlets was calculated overall, and by local authority and Scottish Index of Multiple Deprivation (SIMD) quintile. Population density for each local authority was added using 2022 census data (Scottish Government Scotland's Census, 2022). SIMD is a relative measure of deprivation across 6976 small areas (called data zones), taking into account information across seven domains: income, employment, education, health, access to services, crime, and housing (Scottish Government, 2020). Look-up tables which relate individual postcodes to data zones were used to link SIMD ranks to each food establishment (Scotland NRo, 2025). A total of 732 (2 %) food outlets were excluded from the analysis by SIMD as the postcode specified in the FSA database was not available in the lookup tables. Differences in the proportion of OOH, food retail versus other outlets between quintiles of neighborhood deprivation, overall and stratified by local authority, were evaluated using chi-square tests. Twenty-one of thirty-two local authorities had fewer than five outlets in a given SIMD quintile and were excluded from the analysis. To assess the association between deprivation and total outlet density, we employed a Poisson regression model, with SIMD quintile as the independent variable and outlet density as the dependent variable. All analyses were done in R version 4.2 (R Core Team, 2022).

## 3 Results

Overall, in Scotland, 59 % (*n* = 18,409) of food outlets were OOH, 28 % (*n* = 8757) retail, and 13 % (*n* = 3969) other. “Restaurant/café/canteen” was the most common food outlet type (*n* = 9248, 30 %), followed by “Retailers – other” (*n* = 6881, 22 %), and “Takeaway/sandwich shops” (*n* = 5430, 17 %) (**Supplementary Material S2**). “Retailers - supermarkets/hypermarkets” accounted for only 6 % (*n* = 1876) of food outlets.

### 3.1 Differences by local authority

The proportion of food outlet types varied by local authority, with densely populated areas having a higher number of outlets as well as outlet density. Glasgow City had the highest number of all types of food outlets (*n* = 4977) including 65 % OOH and 23 % retail. The City of Edinburgh had the highest number of pubs/bars (*n* = 524, 15 % of total outlets) and Highlands had the highest number of home caterers/charities (*n* = 1016, 39 % of total outlets). Shetland Islands, Comhairle nan Eilean Siar (Western Isles), and Orkney Islands had fewer than 200 total food outlets (**Supplementary Material S3**).

Out of 32 local authorities, 30 had more than 50 % of their food outlets classified as OOH (**Supplementary Material S6)**. Aberdeenshire and Highlands were the two exceptions where 41 % and 27 % of food outlets were OOH, respectively. The City of Edinburgh (78 %) had the highest proportion of OOH outlets, followed by Argyll and Bute (77 %). Shetland Islands (40 %) had the highest proportion of retail outlets, followed by Comhairle nan Eilean Siar (Western Isles) (39 %), West Dunbartonshire (37 %) and Inverclyde and North Ayrshire (both 36 %). Highlands was the only local authority with an equally low proportion of both OOH (27 %) and retail outlets (28 %); a majority of outlets were other (45 %).

On average, the OOH outlet density was nearly double that of retail in Scotland: 1.9 OOH outlets per km^2^ versus 0.8 retail outlets per km^2^. Local authorities with a high population density such as Glasgow City, Dundee City, City of Edinburgh, and Aberdeen City had the highest OOH and retail densities (Table 2). Highlands, Argyll and Bute, Shetland Islands, and Comhairle nan Eilean Siar (Western Isles) had the lowest OOH and retail density of less than one outlet per km^2^.

**Table 2:** Population density (People per km^2^), percentage of data zones in the most deprived Scottish Index of Multiple Deprivation quintile, and density of total food outlets, out of home, retail, and other food outlets (outlets per km^2^), by local authority in Scotland, 2024 (*n* = 31,135).

Local Authority	Population Density^1^ (People per km^2^)	Most deprived data zones^2^ (%)	Outlet Density (Total Outlets per km^2^)	Out of home Density (Outlets per km^2^)	Retail Density (Outlets per km^2^)	Other^3^ Density (Outlets per km^2^)
Glasgow City	3555	45	28.5	18.5	6.4	3.6
Dundee City	2477	37	16.5	10.2	4.4	1.8
Edinburgh (City of)	1947	12	13.1	10.3	2.2	0.6
Aberdeen City	1207	10	7.5	4.6	2.2	0.7
North Lanarkshire	726	34	3.2	1.8	1.1	0.3
Renfrewshire	703	25	3.6	2.2	1.1	0.3
East Dunbartonshire	625	4	2.2	1.3	0.7	0.2
West Dunbartonshire	557	40	2.7	1.6	1	0.1
East Renfrewshire	556	6	1.1	0.6	0.4	0.1
Falkirk	533	16	2.4	1.5	0.8	0.1
Inverclyde	489	45	2.2	1.3	0.8	0.1
West Lothian	424	15	0.9	0.6	0.3	< 0.1
Clackmannanshire	325	25	1.9	1	0.6	0.2
Fife	280	20	1.8	1	0.6	0.2
Midlothian	273	9	1.0	0.7	0.3	< 0.1
South Lanarkshire	185	20	0.9	0.6	0.3	0.1
East Lothian	165	6	0.7	0.4	0.2	0.1
North Ayrshire	151	40	0.8	0.5	0.3	0.1
East Ayrshire	95	31	0.5	0.3	0.2	< 0.1
South Ayrshire	91	18	0.6	0.4	0.2	< 0.1
Angus	52	8	0.3	0.2	0.1	< 0.1
Aberdeenshire	42	3	0.2	0.1	0.1	< 0.1
Moray	42	3	0.2	0.1	0.1	< 0.1
Stirling	42	12	0.2	0.1	0.1	< 0.1
Perth and Kinross	29	6	0.1	0.1	< 0.1	< 0.1
Scottish Borders	25	6	0.1	0.1	< 0.1	< 0.1
Dumfries and Galloway	23	9	0.1	0.1	< 0.1	< 0.1
Orkney Islands	22	0	0.2	0.1	0.1	< 0.1
Shetland Islands	16	0	0.1	< 0.1	< 0.1	< 0.1
Argyll and Bute	13	10	< 0.1	< 0.1	< 0.1	< 0.1
Highlands	9	10	0.1	< 0.1	< 0.1	< 0.1
Comhairle nan Eilean Siar (Western Isles)	9	0	0.1	< 0.1	< 0.1	< 0.1
**Average**	**689**	**20**	**0.4**	**1.9**	**0.8**	**0.3**

Local authorities are sorted from highest to lowest population density.

1 https://www.scotlandscensus.gov.uk/2022-results/scotland-s-census-2022-rounded-population-estimates/

2 https://www.gov.scot/publications/social-capital-community-wellbeing-scotland/pages/13/

3 “Other” includes home caterers, charitable/community organizations, and mobile caterers.

### 3.2 Differences by neighborhood deprivation

**Fig. 2:**
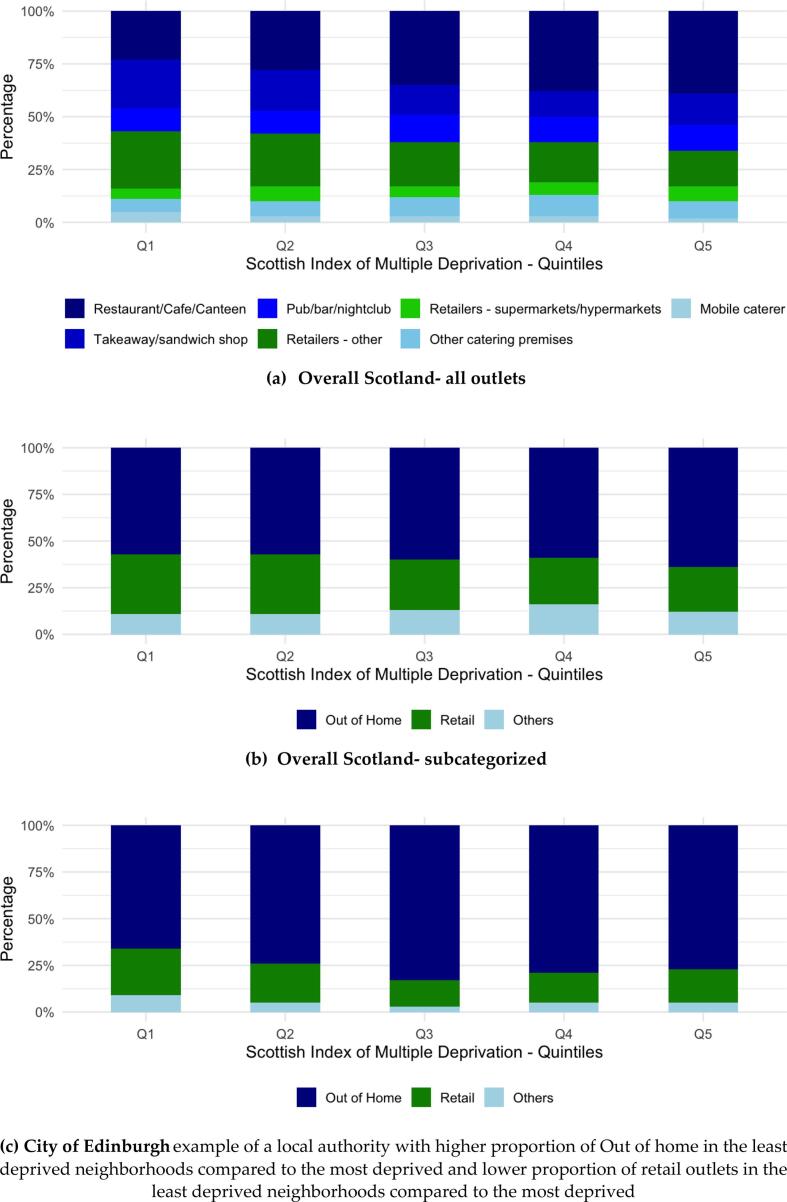

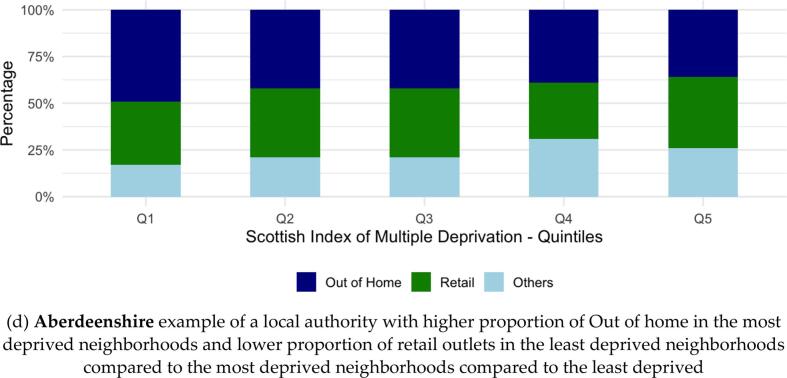
Association of Scottish Index of Multiple Deprivation with classification of food outlets in Scotland, 2024.

Overall, the most deprived areas had significantly higher total outlet density compared to all other less deprived quintiles (comparing 1st quintile (ref) and 5th SIMD quintile β = −0.31, SD = 0.01, *p* < 0.01) [**Supplementary Material S4]**. The proportion of OOH outlets was higher in the least deprived neighborhoods compared to the most deprived (65 % versus 58 %, p < 0.01) [Fig. 2**, Supplementary Material S5]**. This was driven by a higher proportion of “Restaurants/cafés/canteens” (37 % of total food outlets in the least deprived neighborhoods versus 23 % in the most deprived). In contrast, “Takeaways/sandwich shops” comprised a smaller proportion in the least deprived neighborhoods (16 % versus 24 % in the most deprived).

The proportion of food retail outlets was lower in the least deprived neighborhoods compared to the most deprived (24 % versus 32 %, respectively, p < 0.01) [Fig. 2**, Supplementary Material S5**]. This was driven by a lower proportion of “Retailers - other” (outlets primarily selling non-food items with a limited food selection, 16 % in the least deprived neighborhoods versus 26 % in the most deprived neighborhoods). In contrast, the proportion of “Retailers - supermarkets/hypermarkets” was higher in the least deprived neighborhoods compared to the most deprived (7 % versus 6 %).

Five local authorities, namely, Aberdeen City, Fife, Glasgow City, City of Edinburgh and Stirling followed the same trend as in Scotland overall. However, the opposite was true in Aberdeenshire*,* Renfrewshire and South Lanarkshire, where the proportion of OOH was higher in the most deprived neighborhoods and the proportion of retail outlets was lower in the most deprived neighborhoods*.* City of Edinburgh and Aberdeenshire shown as examples in Fig. 2, the rest shown in **Supplementary Material S6**). In Perth and Kinross, the most deprived neighborhoods had a significantly greater proportion of both OOH and retail outlets than the least deprived neighborhoods. North Lanarkshire had the same proportion of OOH outlets in the most and least deprived neighborhoods but a higher proportion of retail outlets in the most deprived neighborhoods.

## 4 Discussion

This first comprehensive analysis of Scotland's food environment, covering 31,135 registered food businesses, found that 60 % were OOH, with restaurants/cafés/canteens accounting for half of these outlets. Across all deprivation levels, OOH outlets outnumbered supermarkets and other food retailers, but their makeup varied: takeaways were more common in deprived areas, while restaurants and cafés were more common in affluent ones. Several local authorities had fewer than one food retail outlet per km^2^, and some of these had a high proportion of “Other” outlets, such as, home caterers and charitable/community organizations, highlighting potentially unique aspects of food environments in these areas of Scotland where limited food retail access appears to be supplemented by informal or community-based sources.

It is understandable that retail outlets are less numerous than OOH outlets, as the area occupied by a single large supermarket could accommodate several OOH outlets. Nevertheless, these results highlight a significant opportunity to improve public health through the OOH sector, as proposed in the Scottish Government's *Eating Out, Eating Well* framework. The OOH sector varied by local authority and deprivation. For example, consistent with one previous study (Sauveplane-Stirling et al., 2014), we found an especially high density of OOH in Glasgow City. Takeaways/sandwich shops were more prevalent in the most deprived areas compared to the least deprived. This is consistent with a recent analysis in England of the same FSA database which found fast food outlets per 100,000 population in the most deprived decile of lower tier local authorities were double the level in the least deprived decile (Disparities, 2025).

Beyond OOH, other aspects of the food environment varied by local authority. In particular, the Highlands was the only local authority where the informal food economy, including home caterers, village halls, and community centers, comprised the largest share (46 %) of food outlets. This contrasts with other local authorities such as Dundee City and Glasgow City where 99 % of people reside in large urban areas and have a higher number and proportion of both OOH and retail food outlets. These results align with other studies conducted in remote rural areas in Scotland which found that populations access food through various channels, such as crofts, community co-operatives, social enterprises, community gardens, and food banks (Freathy et al., 2024). Grocery provision is limited outside the main towns in these areas (Calderwood and Freathy, 2014). Our analysis highlighted that while the Comhairle nan Eilean Siar (Western Isles), Orkney Islands, and Shetland Islands did not have a high proportion of “Other” outlets compared to the Highlands, they had very few outlets overall, which may result in poor food accessibility.

While this is the first study of all registered food businesses in Scotland using the latest FSA data, it has limitations. While we were able to capture some level of specificity within the food sector in terms of business classification, a more granular classification of food outlets (e.g., bakeries, fish and chips) would provide a more complete picture of the food environment. Self-selecting business categories and manual cleaning for others may have introduced some measurement error. Further, OOH may be underestimated by not including mobile caterers. This research does not take into account the digital food environment, including online delivery options from both OOH and food retail outlets, or “dark kitchens” which rent kitchens to many delivery-only restaurants. Future studies could include information on whether businesses offer delivery services, as this may impact food access. Data were downloaded on 1st February 2024, but it is possible that some of the food businesses permanently closed or new food businesses have registered since that date. While collecting primary data on food outlets is ideal, such “ground truthing” is resource and time intensive. Thus, secondary data plays a crucial role in quantifying food environments. Some studies have addressed the accuracy and reliability of secondary food outlet data sources in relation to their utility for use in food environment research in the UK and found it useful for surveillance of the food environment (Lake et al., 2012). An evaluation of the validity and spatial accuracy of the FSA database in England demonstrated high reliability, with a positive predictive value of 95 % and a sensitivity of 89 % (Kirkman et al., 2021), suggesting the results presented herein are likely to be reasonably accurate.

This study highlights improvements to both the OOH and retail food environment are necessary. The Scottish Government proposals to restrict promotion of high fat, sugar, and salt (HFSS) foods in retail and OOH settings aim to tackle this issue, following similar measures introduced in England in 2022 (Public, 2021; Scottish Goverment, 2022). However, early qualitative analysis shows uneven implementation, highlighting the need for complementary policies to improve the accessibility, affordability, and promotion of healthier options (Muir et al., 2023). Despite this, the legislation could reduce impulse HFSS purchases creating healthier retail environments for consumers (Muir et al., 2023). A recent longitudinal analysis found that fast food outlet density has grown faster than supermarkets in Great Britain, especially in deprived areas (Boskovic et al., 2025). Changes to neighborhood planning and licensing of food premises are needed to put food environments on a new trajectory, future proofing further growth in food outlets primarily selling unhealthy food. Takeaway management zones offer one. An evaluation of local authorities in England who adopted takeaway management zones found 0.83 fewer new outlets opened per local authority than expected without such policies (Rahilly et al., 2024a) and improve health by reducing obesity and related diseases (Rogers et al., 2024). However, studies also state limitations like such policies may regulate *future* growth but do not address the existing high density of takeaway outlets, particularly in deprived areas (Brown et al., 2021) or ‘grab and go’ type of foods which are the main form of OOH food in Scotland (Clodagh Elrick NFaLW, 2024). Evidence also suggests that they are most effective when implemented as full management zones, compared with time management zones, which only restrict opening hours for new outlets (Rahilly et al., 2024b). However, restricting access to unhealthy options must be accompanied by parallel efforts to promote and improve access to affordable healthier alternatives. Our study offers insights to inform stakeholder discussions on improving both the OOH and retail sectors across Scotland.

## 5 Conclusions

This analysis of Scotland's food environment revealed that though OOH outlets far outnumber retail in all of Scotland, unique food environments exist in different local authorities. These insights can guide local development, and tailored strategies can be proposed to effectively improve the healthfulness of food environments.

## CRediT authorship contribution statement

**Deksha Kapoor:** Conceptualization, Methodology, Formal analysis, Writing – original draft. **Joe Kennedy:** Software (AI), Methodology, Data curation, Writing – review and editing. **Kirsteen Shields:** Conceptualization, Writing – review and editing. **Christian Reynolds:** Conceptualization, Writing – review and editing. **Tom Clemens:** Data curation, Conceptualization, Methodology, Writing – review and editing. **Lindsay M. Jaacks:** Conceptualization, Methodology, Validation, Supervision, Writing – review and editing.

## Informed consent statement

Not applicable. This study is based on publicly available food outlet data and is exempt from human subjects' ethics committee review.

## Funding

This research did not receive any specific grant from funding agencies in the public, commercial, or not-for profit sector.

## Declaration of competing interest

The authors declare the following financial interests/personal relationships which may be considered as potential competing interests: CR has advisory positions on boards at the Nutrition Society (Food systems theme lead) and the Institute of Food Science & Technology (Sustainability working group). He is part of the Sustainable Diet Working Group, Faculty of Public Health, and the British Standards Institution/ International Organization for Standardization committee ISO/TC 34/SC 20 (Food loss and waste). CR has received payment via City, University of London for consulting for: WRAP (a UK NGO); Zero Waste Scotland; DEFRA and the FSA (UK government). CR has discussed his research in expert interviews or as part of an expert advisory group (for no fee/ Pro Bono) with the following organizations: Collider Lab, YUM Brands (2020); Fwd. (2020); Greener Beans (2020); QUT Digital Media Research Centre (2020); Haier Israel Innovation Center, Ltd. (2021); Almond Board of California, via Porter Novelli (2022). CR has carried out Pro-bono grant reviews for the Fruit Juice Science Centre (2023). CR has been paid a Speaker's Stipend by the following events: The Folger Institute (2020). CR has chaired panels and has presented at the following organizations (for no fee/Pro Bono): British Nutrition Foundation (2023); Nutrilicious (2022/2023/2024); MyNutriWeb (2022/2023/2024); Nutrition Society (2022/2023/2024); Aprifel (2018/2023). CR is a member of the EGEA Scientific Committee and co-chair of a session of the EGEA conference (2023). This has meant his registration and flight/accommodation have been paid for by Aprifel. CR has won competitive research funding (€49,858) from the following independent foundation: The Alpro Foundation (2020). DK, JK, KS, TC, and LMJ have no conflicts of interest to declare.

## Data Availability

The dataset and R scripts used in this study are available from https://github.com/Deksha1/Scotland-Food-Environment

## Appendix A Supplementary data

Supplementary material Image 1
