# Supplementary material for: Characterizing the food environment in Scotland and its association with deprivation: A national study
Source: Prev Med Rep. 2025 Sep 23;59:103254. doi: 10.1016/j.pmedr.2025.103254 (PMC12510213; doi:10.1016/j.pmedr.2025.103254)
Supplement: Supplementary file 1 — Supplementary material [file mmc1.docx]

**Table of Contents**

| **Table no.** | **Title** | **Page No** |
| --- | --- | --- |
| Table S1 | Use of Open-AI’s GPT-4 large language model for categorizing food outlets in Scotland, 2024. | 2 |
| Table S2 | Classification of food outlets in Scotland, 2024 (n=31,135). | 3 |
| Figure S3 | Distribution (counts and proportions) of food outlets by local authority in Scotland, 2024 (n=31,135). | 3-4 |
| Table S4 | Association of density of food outlets (area per km^2^) and by Scottish Index of Multiple Deprivation quintile in Scotland, 2024. | 4 |
| Table S5 | Food outlet classification, overall and by Scottish Index of Multiple Deprivation quintile in Scotland, 2024. | 5 |
| Table S6 | Food outlet classification by local authority and Scottish Index of Multiple Deprivation in Scotland, 2024. | 6-10 |

**Table S1: Use of Open-AI’s GPT-4 large language model for categorizing food outlets in Scotland, 2024**

| Prompt used for identifying businesses to exclude from the list of food businesses | *“Use your extensive domain knowledge of businesses in Scotland to go through the list {LIST OF OUTLETS} and assess whether it engages in the direct sale of food and/or drink to consumers such as butchers, newsagents, grocery stores, coffee shops, supermarkets, restaurants, cafes, bakeries, butchers, fishmongers and similar outlets. If the business DOES NOT meet these criteria, such as a village hall or a catering company, include it in a new list of companies that do not primarily sell food and drink. Only output the new list. Do not include any other text in the output.”* |
| --- | --- |
| List of businesses hallucinated by the model, i.e., those businesses that were not included in the original list of businesses and were generated by GPT-4 (n=34) | 'A And M Catering - Street Trader'  'Delta Facility',  'ELIOR @ BABCOCK (Terminal 2)',  'Glasgowean Health Shop',  'GLOW With Gayle',  'Govan Community Fire Station',  'GRANITE CITY (Landside)',  'HALFORDS (RETAIL SHOP ONLY)',  'HAZLEHEAD GOLF CLUB (Bar only)',  'Hill Of Banchory School',  'Hobbycraft',  'J and JG Dickson And Son Ltd',  'Linkes (SCIO)',  'MacKays Ltd. Retail Shop',  'Maitland Bowling Club',  'Motor Fuel Group Ltd (Grantown))',  'NATURES WAY (CHINESE HERBAL MEDICINE)',  'Option One Events',  'Piazza News',  'Pollok House',  'Reliance @ ABDN SHERIFF COURT (CELL AREA',  'SCAMPS (AFTER SCHOOL CLUB)',  'SODEXO @ SCOTTISH GOVERNMENT (MARINE LAB)',  'SODEXO @ SHELL (WOODBANK)',  'ST MARYS (SHARED) CHURCH',  'SUPERDRUG (UNIT 15)',  'THE 1224 CLUB (MASONIC LODGE)',  'The Balloon And Chocolate Company . . .',  'The KEY',  'The Tunnels',  'W H SMITH (AIRSIDE)',  'W H SMITH (LANDSIDE)',  'W H SMITH (LANDSIDE) (ARRIVALS)',  'Woodend Bowling Club'] |
| Type of hallucinations by the model (examples) | 1. Generated ‘A And M Catering - Street Trader' from the original A and M Catering - Street Trader by changing case 2. Generated "ELIOR @ BABCOCK [Terminal 2]" from the original ELIOR @ BABCOCK (Terminal 2) by changing brackets 3. Generated 'Glasgowean Health Shop' from the original "Glaswegian Health Shop" by misspelling. |

**Table S2. Classification of food outlets in Scotland, 2024 (n=31,135).**

| Food outlet type | N (%) |
| --- | --- |
| Restaurant/Cafe/Canteen | 9,248 (30) |
| Takeaway/sandwich shop | 5,430 (17) |
| Pub/bar/nightclub | 3,731 (12) |
| Subtotal out of home | **18,409 (59)** |
| Retailers - supermarkets/hypermarkets | 1,876 (6) |
| Retailers - other | 6,881 (22) |
| Subtotal food retail | **8,757 (28)** |
| Other catering premises | 2,803 (9) |
| Mobile caterer | 1,166 (4) |
| Subtotal other | **3,969 (13)** |

**Figure S3. Distribution (counts and proportions) of food outlets by local authority in Scotland, 2024 (n=31,135).**

**
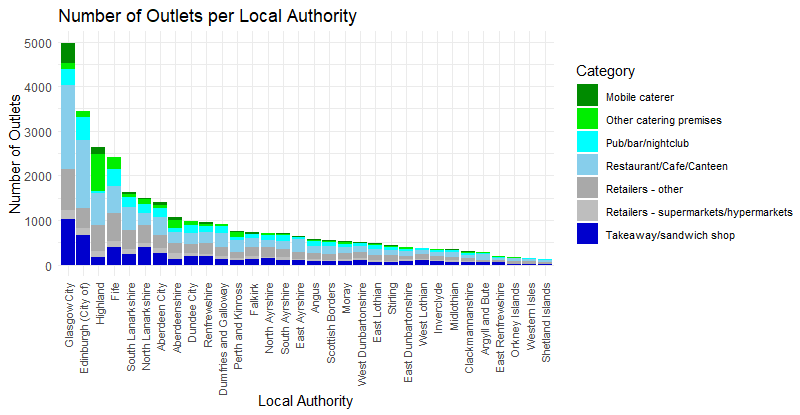
**

1. **Frequency of food outlets**

**
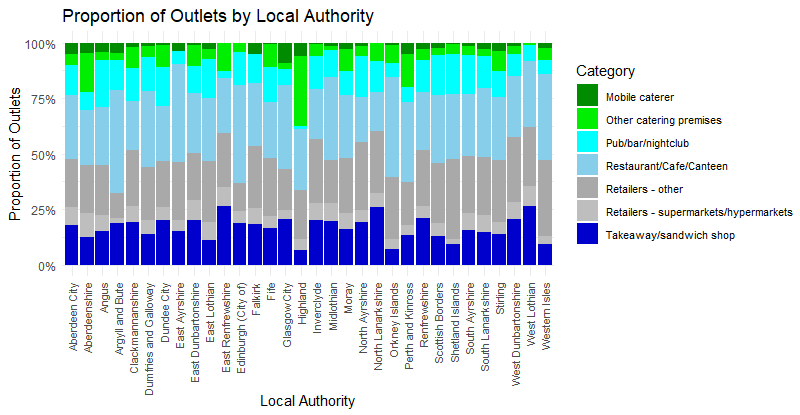
**

**(b) Proportion of outlets**

**Table S4: Association of density of total food outlets (area per km^2^) and by Scottish Index of Multiple Deprivation quintile in Scotland, 2024.**

| **SIMD Quintile** | **β** | **SE** | **p-value** |
| --- | --- | --- | --- |
| Quintile 1 (ref) | 1.00 |  | – |
| Quintile 2 | -0.13 | 0.01 | <0.01 |
| Quintile 3 | -0.30 | 0.01 | <0.01 |
| Quintile 4 | -0.52 | 0.01 | <0.01 |
| Quintile 5 | -0.31 | 0.01 | <0.01 |

**Table S5: Food outlet classification, overall and by Scottish Index of Multiple Deprivation quintile in Scotland, 2024.**

| **Characteristic** | **Overall**  N = 30,403^1^ | **1**  N = 7,251^1^ | **2**  N = 7,570^1^ | **3**  N = 6,621^1^ | **4**  N = 5,652^1^ | **5**  N = 3,309^1^ | **p-value**^2^ |
| --- | --- | --- | --- | --- | --- | --- | --- |
| **Business type** |  |  |  |  |  |  | <0.01 |
| Mobile caterer | 1,027 (3%) | 337 (5%) | 229 (3%) | 209 (3%) | 170 (3%) | 82  (2%) |  |
| Other catering premises | 2,728 (9%) | 451 (6%) | 616 (8%) | 669 (10%) | 697 (12%) | 295 (9%) |  |
| Pub/bar/nightclub | 3,658 (12%) | 821 (11%) | 885 (12%) | 868 (13%) | 698 (12%) | 386 (12%) |  |
| Restaurant/Cafe/Canteen | 9,062 (30%) | 1,641 (23%) | 2,020 (27%) | 2,158 (33%) | 2,005 (35%) | 1,238 (37%) |  |
| Retailers - other | 6,747 (22%) | 1,884 (26%) | 1,853 (24%) | 1,384 (21%) | 1,081 (19%) | 545 (16%) |  |
| Retailers - supermarkets/hypermarkets | 1,838 (6%) | 402 (6%) | 506 (7%) | 354 (5%) | 339 (6%) | 237 (7%) |  |
| Takeaway/sandwich shop | 5,343 (18%) | 1,715 (24%) | 1,461 (19%) | 979 (15%) | 662 (12%) | 526 (16%) |  |
| **Food outlet classification** |  |  |  |  |  |  | <0.01 |
| Out of home | 18,063 (59%) | 4,177 (58%) | 4,366 (58%) | 4,005 (60%) | 3,365 (60%) | 2,150 (65%) |  |
| Other | 3,755 (12%) | 788 (11%) | 845 (11%) | 878 (13%) | 867 (15%) | 377 (11%) |  |
| Retail | 8,585 (28%) | 2,286 (32%) | 2,359 (31%) | 1,738 (26%) | 1,420 (25%) | 782 (24%) |  |
| ^1^% (n) ^2^Pearson's Chi-squared test | | | | | | | |

**Table S6: Food outlet classification by local authority and Scottish Index of Multiple Deprivation in Scotland, 2024.**

| **Local authorities** | **Out of home**  **N (%)** | **Retail**  **N (%)** | **Other**  **N (%)** | **Grand Total** | **p-value^1^** |
| --- | --- | --- | --- | --- | --- |
| **SIMD quintile** |  |  |  |  |  |
| **Aberdeen City** | 845 (61%) | 139 (10%) | 412 (30%) | 1396 | 0.03 |
| 1 | 58(55) | 10(9) | 38(36) | 106 |  |
| 2 | 226(60) | 29(8) | 123(33) | 378 |  |
| 3 | 283(64) | 54(12) | 104(24) | 441 |  |
| 4 | 86(59) | 10(7) | 51(35) | 147 |  |
| 5 | 178(60) | 30(10) | 90(30) | 298 |  |
| **Aberdeenshire** | 433 (41%) | 282 (27%) | 346 (33%) | 1061 | 0.03 |
| 1 | 59(49) | 20(17) | 41(34) | 120 |  |
| 2 | 39(42) | 19(21) | 34(37) | 92 |  |
| 3 | 104(42) | 51(21) | 92(37) | 247 |  |
| 4 | 113(39) | 89(31) | 86(30) | 288 |  |
| 5 | 70(36) | 51(26) | 74(38) | 195 |  |
| **Angus** | 356 (63%) | 48 (8%) | 165 (29%) | 569 | sample size too small |
| 1 | 46(69) | 4(6) | 17(25) | 67 |  |
| 2 | 117(60) | 11(6) | 68(35) | 196 |  |
| 3 | 98(59) | 13(8) | 54(33) | 165 |  |
| 4 | 70(63) | 16(14) | 26(23) | 112 |  |
| 5 | 17(71) | 2(8) | 5(21) | 24 |  |
| **Argyll and Bute** | 221 (77%) | 25 (9%) | 40 (14%) | 286 | sample size too small |
| 1 | 67(89) | 2(3) | 6(8) | 75 |  |
| 2 | 54(86) | 3(5) | 6(10) | 63 |  |
| 3 | 43(61) | 14(20) | 14(20) | 71 |  |
| 4 | 45(76) | 6(10) | 8(14) | 59 |  |
| 5 | 5(56) | 0(0) | 4(44) | 9 |  |
| **Clackmannanshire** | 162 (55%) | 36 (12%) | 96 (33%) | 294 | sample size too small |
| 1 | 72(52) | 19(14) | 48(35) | 139 |  |
| 2 | 43(55) | 5(6) | 30(38) | 78 |  |
| 3 | 17(55) | 5(16) | 9(29) | 31 |  |
| 4 | 12(63) | 4(21) | 3(16) | 19 |  |
| 5 | 17(65) | 2(8) | 7(27) | 26 |  |
| **Comhairle nan Eilean Siar (Western Isles)** | 81 (52%) | 14 (9%) | 60 (39%) | 155 | sample size too small |
| 2 | 32(60) | 6(11) | 15(28) | 53 |  |
| 3 | 46(46) | 8(8) | 45(45) | 99 |  |
| **Dumfries and Galloway** | 583 (64%) | 61 (7%) | 272 (30%) | 916 | sample size too small |
| 1 | 145(73) | 3(2) | 51(26) | 199 |  |
| 2 | 149(59) | 18(7) | 84(33) | 251 |  |
| 3 | 205(63) | 27(8) | 93(29) | 325 |  |
| 4 | 59(58) | 9(9) | 34(33) | 102 |  |
| 5 | 22(56) | 4(10) | 13(33) | 39 |  |
| **Dundee City** | 612 (62%) | 110 (11%) | 265 (27%) | 987 | sample size too small |
| 1 | 217(62) | 9(3) | 123(35) | 349 |  |
| 2 | 135(48) | 87(31) | 57(20) | 279 |  |
| 3 | 89(74) | 3(3) | 28(23) | 120 |  |
| 4 | 138(70) | 11(6) | 47(24) | 196 |  |
| 5 | 33(77) | 0(0) | 10(23) | 43 |  |
| **East Ayrshire** | 412 (65%) | 25 (4%) | 194 (31%) | 631 | sample size too small |
| 1 | 139(60) | 12(5) | 82(35) | 233 |  |
| 2 | 112(62) | 3(2) | 66(36) | 181 |  |
| 3 | 96(75) | 5(4) | 27(21) | 128 |  |
| 4 | 40(69) | 1(2) | 17(29) | 58 |  |
| 5 | 21(75) | 4(14) | 3(11) | 28 |  |
| **East Dunbartonshire** | 232 (59%) | 42 (11%) | 117 (30%) | 391 | sample size too small |
| 1 | 16(57) | 3(11) | 9(32) | 28 |  |
| 2 | 93(62) | 13(9) | 45(30) | 151 |  |
| 3 | 5(42) | 3(25) | 4(33) | 12 |  |
| 4 | 40(63) | 9(14) | 15(23) | 64 |  |
| 5 | 77(57) | 13(10) | 44(33) | 134 |  |
| **East Lothian** | 274 (57%) | 38 (8%) | 166 (35%) | 478 | sample size too small |
| 1 | 24(73) | 0(0) | 9(27) | 33 |  |
| 2 | 94(52) | 11(6) | 75(42) | 180 |  |
| 3 | 92(56) | 15(9) | 56(34) | 163 |  |
| 4 | 45(58) | 10(13) | 23(29) | 78 |  |
| 5 | 12(55) | 2(9) | 8(36) | 22 |  |
| **East Renfrewshire** | 100 (53%) | 25 (13%) | 62 (33%) | 187 | sample size too small |
| 1 | 13(54) | 4(17) | 7(29) | 24 |  |
| 2 | 17(41) | 5(12) | 19(46) | 41 |  |
| 3 | 27(59) | 3(7) | 16(35) | 46 |  |
| 4 | 14(54) | 4(15) | 8(31) | 26 |  |
| 5 | 29(58) | 9(18) | 12(24) | 50 |  |
| **Edinburgh (City of)** | 2,703 (78%) | 156 (5%) | 587 (17%) | 3446 | <0.01 |
| 1 | 173(66) | 23(9) | 66(25) | 262 |  |
| 2 | 336(74) | 21(5) | 95(21) | 452 |  |
| 3 | 776(83) | 26(3) | 137(15) | 939 |  |
| 4 | 525(79) | 30(5) | 107(16) | 662 |  |
| 5 | 783(77) | 50(5) | 188(18) | 1021 |  |
| **Falkirk** | 435 (60%) | 40 (6%) | 247 (34%) | 722 | sample size too small |
| 1 | 89(52) | 26(15) | 56(33) | 171 |  |
| 2 | 111(56) | 2(1) | 86(43) | 199 |  |
| 3 | 145(66) | 5(2) | 71(32) | 221 |  |
| 4 | 51(60) | 3(4) | 31(36) | 85 |  |
| 5 | 26(70) | 0(0) | 11(30) | 37 |  |
| **Fife** | 1,359 (56%) | 302 (12%) | 758 (31%) | 2419 | 0.01 |
| 1 | 233(51) | 61(13) | 159(35) | 453 |  |
| 2 | 455(54) | 90(11) | 293(35) | 838 |  |
| 3 | 197(57) | 39(11) | 107(31) | 343 |  |
| 4 | 264(57) | 66(14) | 132(29) | 462 |  |
| 5 | 180(62) | 47(16) | 64(22) | 291 |  |
| **Glasgow City** | 3,234 (65%) | 622 (12%) | 1,121(23%) | 4977 | <0.01 |
| 1 | 1097(57) | 297(15) | 534(28) | 1928 |  |
| 2 | 550(63) | 105(12) | 212(24) | 867 |  |
| 3 | 554(72) | 63(8) | 155(20) | 772 |  |
| 4 | 672(79) | 36(4) | 143(17) | 851 |  |
| 5 | 260(75) | 38(11) | 49(14) | 347 |  |
| **Highland** | 712 (27%) | 1,193(45%) | 732 (28%) | 2637 | <0.01 |
| 1 | 100(29) | 145(42) | 102(29) | 347 |  |
| 2 | 160(27) | 249(42) | 186(31) | 595 |  |
| 3 | 239(27) | 393(45) | 242(28) | 874 |  |
| 4 | 170(24) | 375(52) | 174(24) | 719 |  |
| 5 | 5(9) | 33(62) | 15(28) | 53 |  |
| **Inverclyde** | 201 (58%) | 21 (6%) | 126 (36%) | 348 | sample size too small |
| 1 | 138(57) | 12(5) | 91(38) | 241 |  |
| 2 | 17(57) | 2(7) | 11(37) | 30 |  |
| 3 | 20(65) | 2(6) | 9(29) | 31 |  |
| 4 | 14(61) | 2(9) | 7(30) | 23 |  |
| 5 | 11(52) | 1(5) | 9(43) | 21 |  |
| **Midlothian** | 234 (69%) | 12 (4%) | 93 (27%) | 339 | sample size too small |
| 1 | 31(56) | 2(4) | 22(40) | 55 |  |
| 2 | 85(64) | 3(2) | 44(33) | 132 |  |
| 3 | 50(71) | 3(4) | 17(24) | 70 |  |
| 4 | 45(85) | 3(6) | 5(9) | 53 |  |
| 5 | 21(72) | 2(7) | 6(21) | 29 |  |
| **Moray** | 283 (54%) | 73 (14%) | 171 (32%) | 527 | sample size too small |
| 1 | 19(66) | 3(10) | 7(24) | 29 |  |
| 2 | 59(58) | 8(8) | 34(34) | 101 |  |
| 3 | 58(49) | 23(19) | 37(31) | 118 |  |
| 4 | 88(50) | 27(15) | 60(34) | 175 |  |
| 5 | 30(57) | 8(15) | 15(28) | 53 |  |
| **North Ayrshire** | 411 (58%) | 45 (6%) | 257 (36%) | 713 | sample size too small |
| 1 | 232(58) | 18(5) | 148(37) | 398 |  |
| 2 | 58(55) | 10(10) | 37(35) | 105 |  |
| 3 | 62(55) | 10(9) | 40(36) | 112 |  |
| 4 | 44(57) | 5(6) | 28(36) | 77 |  |
| 5 | 12(57) | 2(10) | 7(33) | 21 |  |
| **North Lanarkshire** | 853 (58%) | 125 (8%) | 503 (34%) | 1481 | 0.01 |
| 1 | 340(58) | 31(5) | 219(37) | 590 |  |
| 2 | 236(55) | 45(10) | 149(35) | 430 |  |
| 3 | 97(58) | 21(13) | 48(29) | 166 |  |
| 4 | 110(56) | 21(11) | 65(33) | 196 |  |
| 5 | 46(58) | 10(13) | 23(29) | 79 |  |
| **Orkney Islands** | 100 (58%) | 16 (9%) | 56 (33%) | 172 | sample size too small |
| 2 | 46(62) | 2(3) | 26(35) | 74 |  |
| 3 | 12(60) | 1(5) | 7(35) | 20 |  |
| 4 | 40(53) | 13(17) | 23(30) | 76 |  |
| 5 | 2(100) | 0(0) | 0(0) | 2 |  |
| **Perth and Kinross** | 422 (55%) | 162 (21%) | 179 (23%) | 763 | <0.01 |
| 1 | 61(70) | 8(9) | 18(21) | 87 |  |
| 2 | 102(63) | 20(12) | 40(25) | 162 |  |
| 3 | 66(48) | 35(26) | 36(26) | 137 |  |
| 4 | 121(50) | 60(25) | 59(25) | 240 |  |
| 5 | 69(52) | 37(28) | 26(20) | 132 |  |
| **Renfrewshire** | 575 (61%) | 80 (8%) | 288 (31%) | 943 | 0.01 |
| 1 | 202(61) | 18(5) | 111(34) | 331 |  |
| 2 | 172(63) | 16(6) | 86(31) | 274 |  |
| 3 | 77(56) | 21(15) | 40(29) | 138 |  |
| 4 | 86(60) | 16(11) | 41(29) | 143 |  |
| 5 | 28(56) | 8(16) | 14(28) | 50 |  |
| **Scottish Borders** | 330 (61%) | 34 (6%) | 176 (33%) | 540 | sample size too small |
| 1 | 25(58) | 2(5) | 16(37) | 43 |  |
| 2 | 132(64) | 9(4) | 66(32) | 207 |  |
| 3 | 83(58) | 8(6) | 51(36) | 142 |  |
| 4 | 47(63) | 6(8) | 22(29) | 75 |  |
| 5 | 29(56) | 6(12) | 17(33) | 52 |  |
| **Shetland Islands** | 71 (55%) | 7 (5%) | 52 (40%) | 130 | sample size too small |
| 2 | 4(44) | 0(0) | 5(56) | 9 |  |
| 3 | 44(56) | 5(6) | 29(37) | 78 |  |
| 4 | 23(55) | 2(5) | 17(40) | 42 |  |
| **South Ayrshire** | 429 (62%) | 40 (6%) | 228 (33%) | 697 | sample size too small |
| 1 | 102(62) | 9(5) | 54(33) | 165 |  |
| 2 | 145(61) | 17(7) | 77(32) | 239 |  |
| 3 | 75(56) | 6(4) | 53(40) | 134 |  |
| 4 | 66(67) | 4(4) | 29(29) | 99 |  |
| 5 | 30(60) | 3(6) | 17(34) | 50 |  |
| **South Lanarkshire** | 994 (61%) | 102 (6%) | 528 (33%) | 1624 | 0.05 |
| 1 | 242(59) | 30(7) | 141(34) | 413 |  |
| 2 | 316(60) | 26(5) | 181(35) | 523 |  |
| 3 | 180(65) | 11(4) | 84(31) | 275 |  |
| 4 | 169(60) | 26(9) | 88(31) | 283 |  |
| 5 | 56(51) | 7(6) | 47(43) | 110 |  |
| **Stirling** | 229 (52%) | 65 (15%) | 149 (34%) | 443 | 0.05 |
| 1 | 38(43) | 17(19) | 33(38) | 88 |  |
| 2 | 16(33) | 10(21) | 22(46) | 48 |  |
| 3 | 31(47) | 11(17) | 24(36) | 66 |  |
| 4 | 105(58) | 18(10) | 57(32) | 180 |  |
| 5 | 31(55) | 10(18) | 15(27) | 56 |  |
| **West Dunbartonshire** | 288 (58%) | 25 (5%) | 184 (37%) | 497 | sample size too small |
| 1 | 115(52) | 11(5) | 94(43) | 220 |  |
| 2 | 74(53) | 9(6) | 56(40) | 139 |  |
| 3 | 69(73) | 4(4) | 22(23) | 95 |  |
| 4 | 14(67) | 0(0) | 7(33) | 21 |  |
| 5 | 11(73) | 1(7) | 3(20) | 15 |  |
| **West Lothian** | 235 (64%) | 4 (1%) | 127 (35%) | 366 | sample size too small |
| 1 | 33(58) | 0(0) | 24(42) | 57 |  |
| 2 | 132(65) | 3(1) | 68(33) | 203 |  |
| 3 | 27(64) | 1(2) | 14(33) | 42 |  |
| 4 | 22(54) | 0(0) | 19(46) | 41 |  |
| 5 | 16(73) | 0(0) | 6(27) | 22 |  |

^1^Pearson's Chi-squared test
